# Human Norovirus NTPase Antagonizes Interferon-β Production by Interacting With IkB Kinase ε

**DOI:** 10.3389/fmicb.2021.687933

**Published:** 2021-07-15

**Authors:** Zifeng Zheng, Yuncheng Li, Mudan Zhang, Yalan Liu, Ming Fu, Sitang Gong, Qinxue Hu

**Affiliations:** ^1^The Joint Laboratory of Translational Precision Medicine, Guangzhou Women and Children’s Medical Center, Guangzhou, China; ^2^The Joint Laboratory of Translational Precision Medicine, Wuhan Institute of Virology, Chinese Academy of Sciences, Wuhan, China; ^3^State Key Laboratory of Virology, Center for Biosafety Mega-Science, Wuhan Institute of Virology, Chinese Academy of Sciences, Wuhan, China; ^4^Savaid Medical School, University of Chinese Academy of Sciences, Beijing, China; ^5^Department of Gastroenterology, Guangzhou Women and Children’s Medical Center, Guangzhou Medical University, Guangzhou, China; ^6^Institute for Infection and Immunity, St George’s, University of London, London, United Kingdom

**Keywords:** human norovirus, NTPase, interferon-β, IkB kinase ε, interferon-regulatory factor-3

## Abstract

Human norovirus (HuNoV) is the leading cause of epidemic acute gastroenteritis worldwide. Type I interferons (IFN)-α/β are highly potent cytokines that are initially identified for their essential roles in antiviral defense. It was reported that HuNoV infection did not induce IFN-β expression but was controlled in the presence of IFN-β in human intestinal enteroids and a gnotobiotic pig model, suggesting that HuNoV has likely developed evasion countermeasures. In this study, we found that a cDNA clone of GII.4 HuNoV, the predominantly circulating genotype worldwide, inhibits the production of IFN-β and identified the viral NTPase as a key component responsible for such inhibition. HuNoV NTPase not only inhibits the activity of IFN-β promoter but also the mRNA and protein production of IFN-β. Additional studies indicate that NTPase inhibits the phosphorylation and nuclear translocation of interferon-regulatory factor-3 (IRF-3), leading to the suppression of IFN-β promoter activation. Mechanistically, NTPase interacts with IkB kinase ε (IKKε), an important factor for IRF-3 phosphorylation, and such interaction blocks the association of IKKε with unanchored K48-linked polyubiquitin chains, resulting in the inhibition of IKKε phosphorylation. Further studies demonstrated that the 1-179 aa domain of NTPase which interacts with IKKε is critical for the suppression of IFN-β production. Our findings highlight the role of HuNoV NTPase in the inhibition of IFN-β production, providing insights into a novel mechanism underlying how HuNoV evades the host innate immunity.

## Introduction

Human norovirus (HuNoV) is a non-enveloped, single-stranded, positive-sense, RNA virus belonging to the caliciviridae family (Thorne and Goodfellow, 2014). The ~7.5 kb genome contains three open reading frames (ORFs) (Lambden et al., 1993). ORF1 encodes a nonstructural polyprotein that is cleaved by its own protease to generate at least six distinct proteins: p48, NTPase, p22, VPg, 3CLpro, and RdRP. ORF2 encodes the major capsid protein VP1, and ORF3 encodes the minor capsid protein VP2, locating inside the viral capsid (Lu et al., 2015). The NTPase protein is cleaved by the viral protease at the 331-696 aa of the ORF1-encoded polyprotein, resulting in a mature protein with a length of 366 aa and a molecular weight of 40 kDa (Belliot et al., 2003). NTPase plays a significant role in viral replication, such as (a) NTP-dependent helicase activity for unrolling of RNA helices, (b) NTP-independent chaperone activity for remodeling the RNA structure and thereby facilitating the annealing of the RNA chains, and (c) collaboration in RNA synthesis carried out by RdRP (Li et al., 2018). Moreover, NTPase, VP1, RdRP, and p48 work coordinately in the HuNoV replication process. VPg plays a role in recruiting cellular translation initiation factors, while p48 and p22 have been reported to interfere with cell signaling pathways (Campillay-Veliz et al., 2020). Norovirus has been subdivided into 10 different genogroups (GI-GX) (Chhabra et al., 2019). Only GI, GII, and GIV infect human and cause acute gastroenteritis (Mulondo et al., 2020). HuNoV is the leading cause of epidemic gastroenteritis worldwide (Bull et al., 2010; Ahmed et al., 2014; Brown et al., 2017), resulting in high morbidity and mortality rates particularly in infants, young children, and the elderly (Campillay-Veliz et al., 2020). Due to the lack of an efficient *in vitro* cell culture system and a suitable animal model, our current understanding of HuNoV infection and pathogenesis is largely limited.

Host innate immune responses induce the production of interferons (IFNs) and pro-inflammatory cytokines (Lee and Min, 2007; Newton and Dixit, 2012; Brubaker et al., 2015). Type I IFN-α/β signaling promotes the production of interferon-stimulated genes (ISGs) and confers fast antiviral protection (Haller et al., 2006). It was reported that IFN-β was effective in suppressing HuNoV replication in HG23 cells harboring a self-replicating HuNoV subgenomic replicon (Chang et al., 2006a). In addition, HuNoV infection did not induce type I IFN responses but was controlled in the presence of IFN-β in the gnotobiotic pig model and human intestinal enteroids (HIEs) (Souza et al., 2007; Jung et al., 2012; Lin et al., 2019, 2020; Hosmillo et al., 2020). Furthermore, a GII.3 HuNoV cDNA clone, which efficiently generates double-stranded RNAs (Qu et al., 2016), commonly as pathogen-associated molecular patterns recognized by cellular sensors (Said et al., 2018), did not induce detectable type I IFN responses but rather inhibited IFN-β production (Qu et al., 2016). All these together suggest that HuNoV has likely developed an unknown mechanism to counteract IFN-β production.

Interferon-regulatory factor-3 (IRF-3) signal pathway is known to play a critical role in innate immune responses for regulating IFN-β production (Sato et al., 1998; Barnes et al., 2002). The retinoic acid-inducible gene I (RIG-I) like receptor (RLR) family functions as major sensors of RNA viruses. Following viral infection, the helicase domain of RIG-I or melanoma differentiation-associated protein 5 (MDA5) of RLR family binds to dsRNA and the recruitment domain binds to mitochondrial antiviral signal protein, which is also known as IPS1 (Kell and Gale, 2015; Esser-Nobis et al., 2020). IPS1 activation enables IkB kinase ε (IKKε) and TANK-binding kinase 1 (TBK1) to phosphorylate and dimerize IRF-3 (Fitzgerald et al., 2003; Fang et al., 2017). IRF-3 translocates from the cytoplasm into the nucleus, resulting in transcription induction of the gene encoding IFN-β (Goubau et al., 2013; Hartmann, 2017). Several viral components from different viruses have been shown to inhibit IFN-β production *via* interfering with IRF-3 signal pathway (Fensterl et al., 2005; Daffis et al., 2007; Paulmann et al., 2008). It is known that RNAs synthesized by transiently expressed HuNoV RdRP can stimulate RIG-I-dependent reporter luciferase production *via* the IFN-β promoter (Subba-Reddy et al., 2011). However, it remains to be determined whether HuNoV modulates IFN-β production through RLR-IRF-3 signal pathway.

In the current study, we demonstrated that a GII.4 HuNoV cDNA clone inhibits IFN-β production and identified the viral NTPase as a key component responsible for such inhibition. We further addressed how HuNoV NTPase inhibits IFN-β production and the key region of NTPase involved in the process. This study reveals a novel mechanism of HuNoV evading innate immunity, providing a basis for further understanding the complexity of HuNoV-host interactions.

## Materials and Methods

### Cell Lines, Viruses, Plasmids, and Abs

Human epithelial cell line Caco-2, HEK 293T cell line, and HeLa cell line were maintained in DMEM (Life Technologies, 11965), containing 100 U/ml penicillin, 100 U/ml streptomycin, and 10% FBS at 37°C in a 5% CO_2_ incubator. Sendai virus (SeV) was propagated in 12-d-old special pathogen-free embryonated eggs (Beijing Merial Vital Laboratory Animal Technology Corporation), and chicken red blood cells were used to measure the titer of SeV by hemagglutination assay (Killian, 2008). Eggs were incubated at 37°C for 12 d before inoculation with SeV. SeV was diluted to 100 hemagglutinating unit (HAU)/ml with FBS-free DMEM. Allantoic cavity of eggs was injected with 300 μl SeV diluent and incubated at 37°C for 72 h. The allantoic fluids were collected after eggs were kept in an incubator at 4°C overnight. Collected virus fluids were briefly centrifuged and stored at −80°C before being used for infection. For hemagglutination assay, equal volume of 1% (v/v) chicken RBCs was mixed with 2-fold serially diluted SeV before added to 96-well V-shaped bottom plates for 45 min at room temperature. The highest dilution of virus that formed diffuse lattice was defined as 1 HAU.

The plasmid pHuNoV containing full-length genome cDNA of GII.4 HuNoV was constructed using an established reverse genetics system as described in the previous study (Katayama et al., 2014). GII.4 HuNoV genes p48, NTPase, VPg, RdRP, NTPase, and its truncations with Flag or HA tag were cloned into the HindIII-BamHI sites of the pcDNA3.1(+) (Invitrogen), using NovoRec PCR one-step directional cloning kit (Novoprotein, NR001) according to the manufacturer’s instructions. P22 and 3CLpro genes of GII.4 HuNoV with a Flag tag were generated and inserted into the pcDNA3.1(+) vector by GenScript, respectively. The pcDNA3-IPS1-Flag expression plasmid, the IFN-β reporter plasmid p125-Luc, and the internal control plasmid phRL-TK were described previously (Cloutier and Flamand, 2010; Li et al., 2010). IRF-3/5D expression plasmid pIRES-hrGFP/IRF-3/5D-Flag (constitutively active mutant of IRF-3) was provided by Dr. Yi-Ling Lin (Graduate Institute of Life Sciences, National Defense Medical Center, Taipei, Taiwan) (Chang et al., 2006b). The reporter plasmid PRD(III-I)_4_-Luc was provided by Dr. Stephan Ludwig (University of Muenster, Muenster, Germany) (Ehrhardt et al., 2004). pEF-Flag-RIG-I-IN (a carboxy-terminally truncated, constitutively active RIG-I mutant) expression plasmid was provided by Dr. Takashi Fujita (Yoneyama et al., 2004). Expression plasmids pcDNA3-TBK1-Flag and pcDNA3-IKKε-Flag were gifts from Dr. Katherine Fitzgerald (University of Massachusetts Medical School, Worcester, MA) (Fitzgerald et al., 2003). All primers used are listed in Supplementary Table S1.

The antibody (Ab) against GII HuNoV capsid was purchased from Invitrogen (MA5-18241). Abs against IRF-3, p-IRF-3, IKKε, and p-IKKε were purchased from Cell Signaling Technology (4302S, 4947S, 2905S, and 8766S). A rabbit Ab against HA tag was purchased from Cell Signaling Technology (3724S), and another mouse Ab against HA tag was purchased from Santa Cruz Biotechnology (sc-7392). Ab against Flag tag was purchased from Sigma-Aldrich (F1804). Ab against β-actin was purchased from Santa Cruz Biotechnology (sc-81178). Ab against proliferating cell nuclear antigen (PCNA) was purchased from Proteintech (10205-2-AP). Alexa Fluor 488-labeled Goat Anti-Mouse IgG (H + L) (A0428) and Alexa Fluor 647-labeled Goat Anti-Rabbit IgG (H + L) (A0468) were from Beyotime. Polyinosinic-polycytidylic acid [Poly(I:C)] was purchased from InvivoGen (tlrl-picwlv).

### Dual Luciferase Report Assay

HEK 293T cells preseeded in 48-well plates overnight were cotransfected with plasmid encoding NTPase, truncated NTPase, or empty vector together with IFN-β reporter plasmid p125-Luc and internal control plasmid phRL-TK. Transfections were performed using Lipofectamine 3,000 (Invitrogen, L3000015) according to the manufacturer’s instructions. At 24 h posttransfection, cells were stimulated with 100 HAU SeV or transfected with Poly(I:C) using Lipofectamine 2000 (Invitrogen, 11668-027) according to the manufacturer’s instructions. The 16 h later, the lysates were harvested from cells and used to measure firefly and renilla luciferase activities with a Dual-Luciferase Reporter Assay System (Promega, E1980) according to the manufacturer’s instructions. For some experiments, NTPase expression plasmid or empty vector, reporter plasmid p125-Luc and phRL-TK, together with plasmid encoding IFN-β pathway inducer RIG-I-IN, IPS1, TBK1, IKKε, or IRF-3/5D was cotransfected into HEK 293T cells for 40 h. The enzymatic activities of firefly and renilla luciferase were measured.

### Real-Time PCR

The NTPase expression plasmid or empty vector was transfected into HEK 293T cells preseeded in 6-well plates. At 24 h posttransfection, cells were stimulated with 100 HAU SeV or Poly(I:C) for 16 h. For some experiments, Caco-2 cells in 6-well plates were transfected with NTPase expression plasmid or empty vector. At 24 h posttransfection, cells were stimulated with IFN-β or IFN-γ for 16 h. In some cases, Caco-2 cells in 6-well plates were transfected with pHuNoV or empty vector. At 24 h posttransfection, cells were stimulated with SeV or transfected with Poly(I:C) for 16 h. TRIzol (Invitrogen, 15596-026) was used to extract total RNA from cells according to the manufacturer’s instructions. The contamination of genomic DNA was eliminated with RNase-free DNase I (Fermentas, EN0521). cDNA was synthesized by SMART MMLV Reverse Transcriptase (Takara, 639522) and used as the template for the amplification of IFN-β, ISGs, or internal GAPDH control. Real-time PCR was performed on an Bio-Rad CFX96 using the iTaq Universal SYBR Green Supermix (Bio-Rad, 1,725,124) according to the following conditions: 95°C for 30 s, followed by 40 cycles of 95°C for 5 s, and 60°C for 30 s. In some cases, Caco-2 cells in 6-well plates were transfected with pHuNoV. At the indicated time posttransfection, TRIzol (Invitrogen, 15596-026) was used to extract total RNA from cells according to the manufacturer’s instructions. Real-time PCR was performed on an Bio-Rad CFX96 using the HiScript ^®^ II One Step qRT-PCR SYBR Green Kit (Vazyme, Q221-01) according to the following conditions: 50°C for 3 min, 95°C for 30 s, followed by 40 cycles of 95°C for 10 s, and 60°C for 30 s. The expression difference was calculated on the basis of 2^-ΔΔ*t*^ values. The primer pairs used are listed in Supplementary Table S2.

### ELISA for IFN-β

Plasmid expressing NTPase, truncated NTPase, or empty vector was transfected into HEK 293T cells preseeded in 6-well plates for 24 h followed by stimulation with 100 HAU SeV for 16 h. For some experiments, Caco-2 cells in 6-well plates were transfected with pHuNoV or empty vector. The 24 h posttransfection, cells were stimulated with SeV or transfected with Poly(I:C) for 16 h. Secreted IFN-β in supernatants was quantified by a human IFN-β ELISA kit (Pierce, 414101) according to the manufacturer’s instructions.

### Western Blotting

Plasmid expressing NTPase, truncated NTPase, or empty vector was transfected into HEK 293T cells preseeded in 6-well plates for 24 h followed by stimulation with 100 HAU SeV for 16 h. Total proteins were released using the Cell Lysis Buffer (Thermo Fisher, 87788). Cytoplasmic and nuclear proteins were isolated using the Nucleus and Cytoplasm Protein Extraction Kit (Beyotime, P0027). For some experiments, Caco-2 cells in 6-well plates were transfected with pHuNoV or empty vector for 24 h. Cell extracts were subjected to 10% SDS-PAGE and transferred to polyvinylidene difluoride membranes (Millipore, 0.45 mm). The membrane was incubated with 5% nonfat milk in TBS-Tween [200 mm NaCl, 0.1% (v/v) Tween 20, 50 mm Tris-HCl (pH 7.5)] for 2 h and then incubated with appropriate primary Abs at room temperature for 2 h. The membrane was washed three times with TBS-Tween followed by incubation with HRP-conjugated goat anti-mouse IgG (Beyotime, A0216) or HRP-conjugated goat anti-rabbit IgG (Beyotime, A0208) at room temperature for 1 h. Following the addition of chemiluminescent substrates (Beyotime, P0018), protein bands were visualized under a FluorChem HD2 Imaging System (α Innotech). For some experiments, blot intensity was quantified using the software Image Lab.

### Immunofluorescence Assay

HeLa cells preseeded in 6-well plates were transfected with plasmid expressing NTPase, truncated NTPase, or empty vector. 24 h later, cells were stimulated with 100 HAU SeV for 16 h. After fixation with 4% paraformaldehyde, cells were permeabilized with 0.2% Triton X-100. Cell were washed three times with PBS and blocked with PBS containing 3% BSA at 4°C overnight. Subsequently, cells were incubated with rabbit anti-human IRF-3 Ab (Cell Signaling Technology, 4302S) and mouse anti-HA polyclonal Ab (Santa Cruz Biotechnology, sc-7392) at a dilution of 1:100 at 37°C for 1 h. After three washes with PBS, cells were then incubated with Alexa Fluor 488-labeled Goat Anti-Mouse IgG (H + L) (Beyotime, A0428) and Alexa Fluor 647-labeled Goat Anti-Rabbit IgG (H + L) (Beyotime, A0468) at a dilution of 1:100 each for 1 h at 37°C. Thereafter, cells were washed three times with PBS and incubated with DAPI (Beyotime, C1005) for 5 min at room temperature. After three washes with PBS, cells were observed under a fluorescence microscope (Nikon, N-STORM).

### RNA Interference

The sequence of small interfering RNA (siRNA) for HuNoV NTPase (5'-CCACTTTAAGAGCTTGTAA-3'), siRNA for tripartite motif-containing 6 (TRIM6) (5'-GCTGCTTCAAGTCCTTGGCTCTGAT-3'), and a siRNA for scrambled negative control sequence were synthesized by the RiboBio. Caco-2 cells were transfected with siRNA for HuNoV NTPase or control siRNA using the riboFECT CP Transfection Kit (RiboBio, C10511-1) according to the manufacturer’s instructions. 24 h posttransfection, cells were transfected with pHuNoV or empty vector for another 24 h. Supernatants were quantified by a human IFN-β ELISA kit (Pierce, 414101) according to the manufacturer’s instructions. TRIzol (Invitrogen, 15596-026) was used to extract total RNA from cells according to the manufacturer’s instructions. cDNA was synthesized by the SMART MMLV Reverse Transcriptase (Takara, 639522) and used to perform real-time PCR. For some experiments, siRNA for HuNoV NTPase or control siRNA was transfected into Caco-2 cells. At 24 h posttransfection, cells were transfected with NTPase-Flag expression plasmid or empty vector for another 24 h. Total protein was isolated. The NTPase expression was confirmed by Western blotting (WB) using anti-Flag Ab. For some experiments, TRIM6-specific siRNA or control siRNA was transfected into HEK 293T cells. At 24 h posttransfection, cells were cotransfected with NTPase-HA expression plasmid or empty vector, reporter plasmid p125-Luc and phRL-TK, together with plasmid encoding IFN-β inducer RIG-I-IN-Flag for another 24 h. The enzymatic activities of firefly and renilla luciferase were measured. Total protein was isolated and the expression of TRIM6, RIG-I-IN-Flag, and NTPase-HA was analyzed by WB using anti-TRIM6 Ab, anti-Flag Ab, and anti-HA Ab, respectively.

### Coimmunoprecipitation Assay

NTPase-HA or truncated NTPase-HA expression plasmid together with IKKε-Flag expression plasmid or empty vector was cotransfected into HEK 293T cells preseeded in 6-well plates. For some experiments, IKKε-Flag expression plasmid together with plasmid expressing NTPase-HA, truncated NTPase-HA, or empty vector was cotransfected into HEK 293T cells preseeded in 6-well plates. 24 h later, cells were stimulated with or without 100 HAU SeV for 16 h. Lysis buffer [150 mm NaCl, 1% NP40, 50 mm Tris (pH 8.0)] containing protease inhibitor mixture (Roche, 11,697,498,001) was used to lysis cells on ice for 10 min. To eliminate the nonspecific binding of other proteins, Dynabeads protein G (Invitrogen, 10003D) was used to pretreat the samples for 2 h at room temperature. 5 μg mouse anti-Flag Ab, 5 μg rabbit anti-HA Ab, or control IgG together with fresh Dynabeads protein G were added to the pretreated samples followed by a rotary incubator at 4°C overnight. After three washes with PBST, the target Ags were subjected to WB analysis after elution by boiling.

### Statistical Analysis

Data analyses were performed with the GraphPad Prism 7 software (GraphPad). Comparison between two groups was analyzed by two-tailed unpaired student t-test. A value of *p* < 0.05 was considered statistically significant.

## Results

### The cDNA Clone of HuNoV Inhibits IFN-β Production

**Figure 1:**
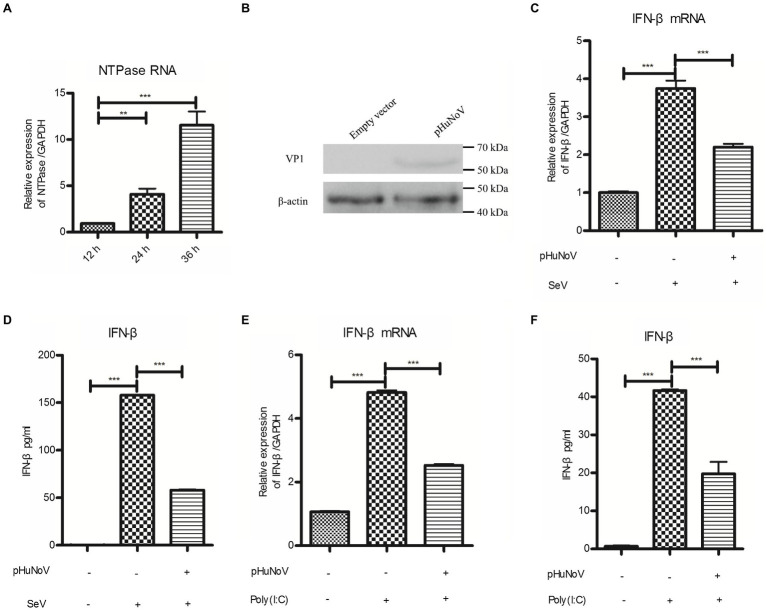
A cDNA clone of human norovirus (HuNoV) inhibits the production of interferons (IFN-β) induced by Sendai virus (SeV) and polyinosinic-polycytidylic acid [Poly(I:C)]. **(A)** Identification of HuNoV RNA expression in cells transfected with a HuNoV cDNA clone. 3 μg pHuNoV was transfected into Caco-2 cells preseeded in 6-well plates. At the indicated time posttransfection, total RNA was extracted from transfected cells to measure NTPase RNA by real-time PCR. **(B)** Identification of HuNoV protein expression in cells transfected with a HuNoV cDNA clone. 3 μg pHuNoV or empty vector was transfected into Caco-2 cells preseeded in 6-well plates for 24 h. Total proteins were isolated. HuNoV VP1 was detected using an anti-HuNoV VP1 Ab, with β-actin being used as a loading control. **(C,D)** A cDNA clone of HuNoV inhibits the production of IFN-β induced by SeV. 3 μg pHuNoV or empty vector was transfected into Caco-2 cells preseeded in 6-well plates. At 24 h posttransfection, cells were stimulated with or without SeV for 16 h. Total RNA was extracted from transfected cells to measure IFN-β mRNA by real-time PCR **(C)**, while IFN-β released in supernatants was determined by ELISA **(D)**. **(E,F)** A cDNA clone of HuNoV inhibits the production of IFN-β induced by Poly(I:C). Caco-2 cells in 6-well plates were transfected with 3 μg pHuNoV or empty vector. 24 h later, cells were transfected with or without 0.5 μg/ml Poly(I:C) for 16 h. Total RNA was extracted from transfected cells to measure IFN-β mRNA by real-time PCR **(E)**, while IFN-β released in supernatants was determined by ELISA **(F)**. One representative experiment out of three is shown for WB. For graphs, data shown are mean ± SD of three independent experiments, with each condition performed in triplicate. ^***^p < 0.001; ^**^p < 0.01.

Due to the lack of an efficient cell culture system *in vitro* and a suitable animal model, the understanding of HuNoV infection and host immune responses to the virus is largely limited. Recently, a GII.3 HuNoV clone constructed using a reverse genetics system has been shown to inhibit IFN-β production in 293FT cells (Qu et al., 2016), highlighting the potential of such system in HuNoV research. Given that the GII.4 genotype is the most prevalent HuNoV in the world and that GII.3 and GII.4 HuNoV may exhibit strain-specific sensitivity to host IFN pathways (Lin et al., 2020), we employed a plasmid pHuNoV containing full-length genome cDNA of GII.4 HuNoV constructed using our established reverse genetics system to address its impact on IFN-β production. The structural proteins of HuNoV can be detected in cells transfected with HuNoV cDNA clone in Caco-2 cells (Oliveira et al., 2018). We first confirmed the expression of HuNoV NTPase RNA and VP1 protein in the cells transfected with GII.4 HuNoV cDNA clone (Figures 1A,B). Caco-2 cells were transfected with pHuNoV or empty vector for 24 and then stimulated with or without SeV for 16 h. Real-time PCR and ELISA were performed to analyze mRNA and protein production of IFN-β, respectively. As shown in Figures 1C,D, the cDNA clone of HuNoV significantly inhibited the mRNA and protein expression of IFN-β induced by SeV. In addition to SeV stimulation, we also conducted experiments under the condition of IFN-β expression induced by Poly(I:C). As shown in Figures 1E,F, the cDNA clone of HuNoV inhibited the mRNA and protein expression of IFN-β induced by Poly(I:C). All these together indicate that the GII.4 HuNoV cDNA clone inhibits IFN-β production.

### HuNoV NTPase Inhibits IFN-β Production

**Figure 2:**
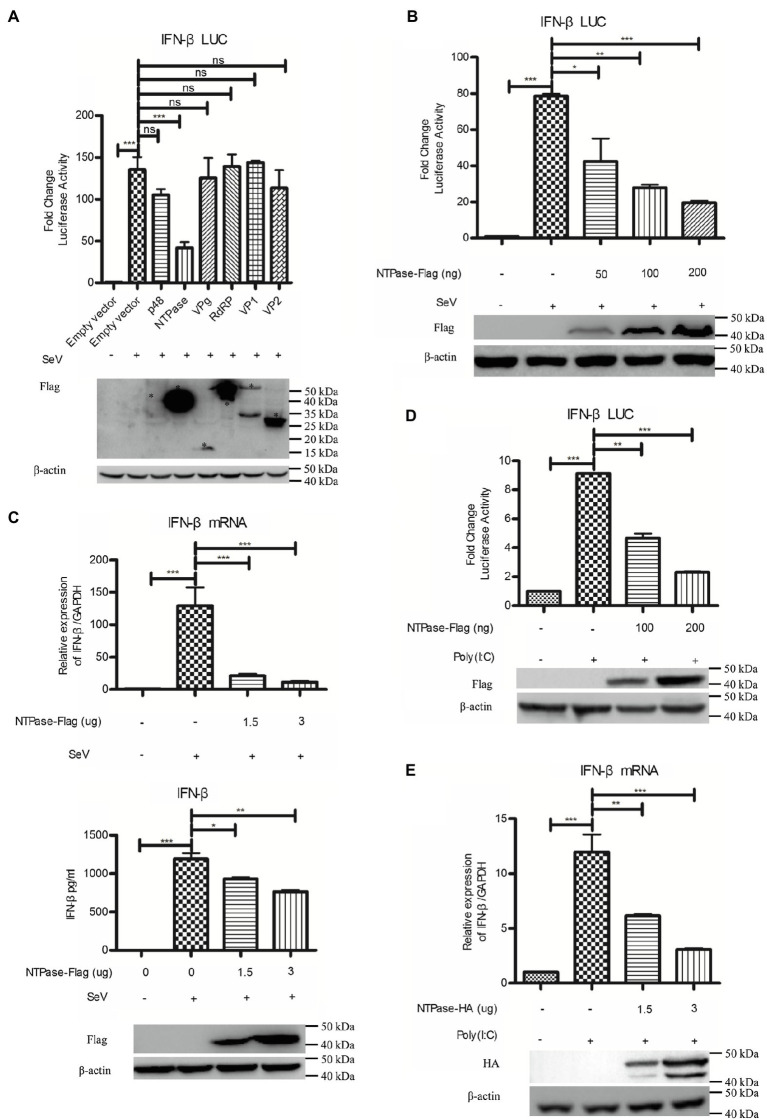
HuNoV NTPase inhibits IFN-β production induced by SeV and Poly(I:C) in a dose-dependent manner. **(A)** Identification of HuNoV proteins that inhibit IFN-β expression. 200 ng plasmid expressing Flag-tagged p48, NTPase, VPg, RdRP, VP1, VP2, or empty vector together with 100 ng p125-Luc and 20 ng phRL-TK was cotransfected into HEK 293T cells preseeded in 48-well plates for 24 h. Cells were stimulated with or without SeV for 16 h. Reporter activities were determined with dual luciferase report (DLR) assay. The expression of HuNoV proteins was analyzed by WB using an anti-Flag Ab. An asterisk indicates HuNoV protein. **(B)** HuNoV NTPase inhibits SeV-induced activation of IFN-β promoter. Various amounts of Flag-tagged NTPase expression plasmid or empty vector together with 100 ng p125-Luc and 20 ng phRL-TK were cotransfected into HEK 293T cells preseeded in 48-well plates for 24 h. Cells were stimulated with or without SeV for 16 h. Reporter activities were determined with DLR assay. **(C)** HuNoV NTPase inhibits the SeV-induced production of IFN-β. HEK 293T cells in 6-well plates were transfected with various amounts of plasmid expressing NTPase or empty vector for 24 h followed by stimulation with or without SeV for 16 h. Total RNA was extracted from transfected cells to measure IFN-β mRNA by real-time PCR, while IFN-β released in supernatants was determined by ELISA. **(D)** HuNoV NTPase inhibits Poly(I:C)-induced activation of IFN-β promoter. HEK 293T cells in 48-well plates were cotransfected with various amounts of Flag-tagged NTPase expression plasmid or empty vector together with 100 ng p125-Luc and 20 ng phRL-TK. At 24 h posttransfection, cells were transfected with or without 0.5 μg/ml Poly(I:C) for 16 h. Reporter activities were determined with DLR assay. **(E)** HuNoV NTPase inhibits the Poly(I:C)-induced production of IFN-β mRNA. HEK 293T cells in 6-well plates were transfected with various amounts of plasmid expressing NTPase or empty vector for 24 h followed by transfection with or without 0.5 μg/ml Poly(I:C) for 16 h. Total RNA was extracted from transfected cells to measure IFN-β mRNA by real-time PCR. The expression of HuNoV NTPase protein was detected using an anti-Flag Ab or anti-HA Ab, with β-actin being used as a loading control. One representative experiment out of three is shown for WB. For graphs, data shown are mean ± SD of three independent experiments, with each condition performed in triplicate. ^***^p < 0.001; ^**^p < 0.01; ^*^p < 0.05; and ns, not significant.

Subsequently, experiments were conducted to address the potential role of HuNoV proteins in inhibiting IFN-β induction. Reporter plasmid p125-luc and phRL-TK together with HuNoV gene (p48, NTPase, p22, VPg, 3CLpro, RdRP, VP1, or VP2) expression plasmid or empty vector were cotransfected into HEK 293T cells. At 24 h posttransfection, cells were stimulated with or without SeV for 16 h. Reporter activities were determined with dual luciferase report (DLR) assay. As shown in Figure 2A; Supplementary Figure S1, HuNoV NTPase and p22, but not p48, VPg, 3CLpro, RdRP, VP1, or VP2, significantly inhibited the activation of IFN-β promoter. Given that the inhibitory effect of NTPase on the IFN-β promoter activity appeared to be stronger than that of p22, we subsequently focused on understanding the mechanism as to how HuNoV NTPase inhibits IFN-β production. To further confirm the effect of NTPase on the activation of IFN-β promoter, HEK 293T cells were cotransfected with reporter plasmid p125-luc and phRL-TK together with various amounts of NTPase expression plasmid for 24 h followed by stimulation with or without SeV for 16 h. As shown in Figure 2B, HuNoV NTPase suppressed the activation of IFN-β promoter in a dose-dependent manner. To confirm the results generated from DLR assay, IFN-β production at the mRNA and protein levels was also analyzed by real-time PCR and ELISA, respectively. As shown in Figure 2C, HuNoV NTPase inhibited the mRNA and protein expression of IFN-β induced by SeV in a dose-dependent manner. In addition to SeV stimulation, we conducted experiments under the condition of IFN-β expression induced by Poly(I:C). p125-Luc and phRL-TK together with plasmid expressing NTPase or empty vector were cotransfected into HEK 293T cells. At 24 h posttransfection, cells were subsequently transfected with or without Poly(I:C) for 16 h to induce IFN-β expression. As shown in Figure 2D, the activation of IFN-β promoter induced by Poly(I:C) was inhibited by HuNoV NTPase. We further assessed IFN-β production induced by Poly(I:C) at mRNA level. HuNoV NTPase significantly inhibited the mRNA expression of IFN-β induced by Poly(I:C) (Figure 2E). Moreover, the HuNoV cDNA clone with NTPase knockdown increased the expression of IFN-β and ISGs (Supplementary Figure S2). These findings together demonstrate that HuNoV NTPase suppresses IFN-β production.

### HuNoV NTPase Suppresses IRF-3 Phosphorylation and Nuclear Translocation

**Figure 3:**
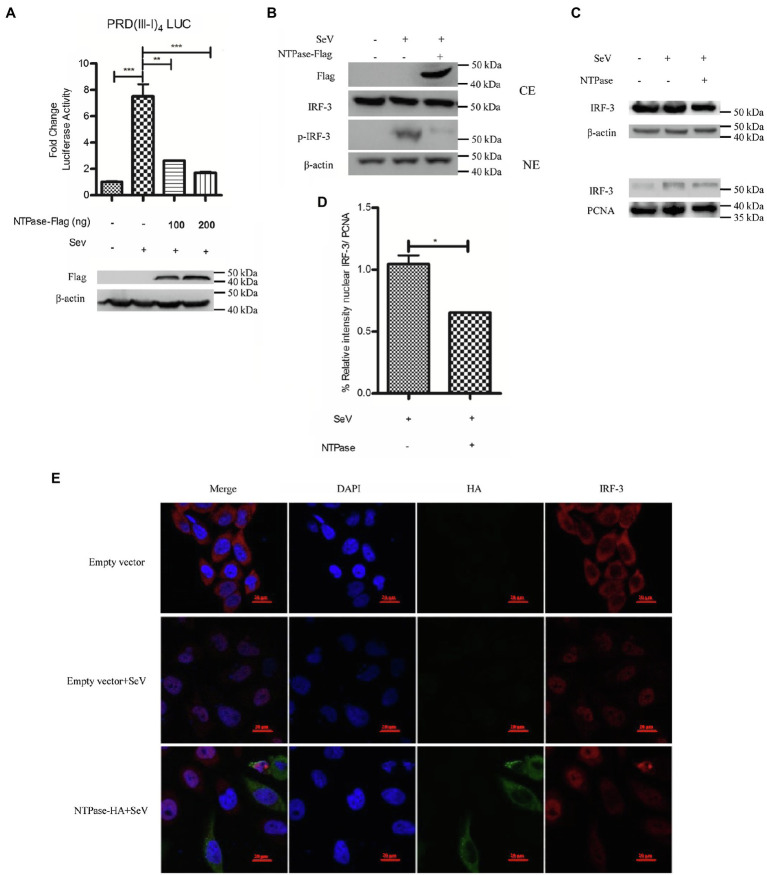
HuNoV NTPase suppresses IRF-3 phosphorylation and translocation. **(A)** HuNoV NTPase inhibits the activation of IRF-3-responsive IFN-β promoter. Various amounts of NTPase-Flag expression plasmid or empty vector together with 100 ng PRD(III-I)_4_-Luc and 20 ng phRL-TK were cotransfected into HEK 293T cells preseeded in 48-well plates for 24 h. Cells were stimulated with or without SeV for 16 h. Reporter activities were determined with DLR assay. **(B)** HuNoV NTPase inhibits the phosphorylation of IRF-3. HEK 293T cells in 6-well plates were transfected with 3 μg plasmid expressing NTPase or empty vector for 24 h followed by stimulation with or without SeV for 16 h. Total protein was isolated. WB was performed to examine IRF-3 and p-IRF-3 using corresponding Abs. The expression of NTPase was monitored using the anti-Flag Ab. **(C)** HuNoV NTPase inhibits IRF-3 nuclear translocation. 3 μg NTPase expression plasmid or empty vector was transfected into HEK 293T cells preseeded in 6-well plates. 24 h later, cells were stimulated with or without SeV for 16 h. Cytoplasmic and nuclear proteins were isolated. IRF-3 levels were measured with anti-IRF-3 Ab. β-actin and PCNA were used as loading controls for cytoplasmic and nuclear proteins, respectively. **(D)** Blot intensity for nuclear IRF-3 was quantified using Image Lab with statistical significance being analyzed from three independent experiments. **(E)** The effect of NTPase on IRF-3 nuclear translocation was determined by indirect IF. HeLa cells in 35-mm dishes were transfected with 3 μg HA-tagged NTPase expression plasmid or empty vector for 24 h and then stimulated with or without SeV for 16 h. Cells were incubated with mouse anti-HA Ab and rabbit anti-IRF-3 pAb, followed by Alexa Fluor 488-labeled Goat Anti-Mouse IgG (H + L) (green) and Alexa Fluor 647-labeled Goat Anti-Rabbit IgG (H + L) (red) as the secondary Abs. Cell nuclei (blue) were stained with DAPI. The images were acquired by fluorescence microscopy using a 60 × objective. One representative experiment out of three is shown. One representative experiment out of three is shown for WB. CE, cytoplasmic protein extract; NE, nuclear protein extract. For graphs, data shown are mean ± SD of three independent experiments, with each condition performed in triplicate. ^***^p < 0.001; ^**^p < 0.01; and ^*^p < 0.05.

Having identified HuNoV NTPase as an important viral protein responsible for IFN-β inhibition, we next assessed the potential signal pathway involved in NTPase-mediated inhibition of IFN-β production. IRF-3 signal pathway is known to have a critical role in viral and bacterial innate immune responses by regulating the production of IFN-β (Sato et al., 1998; Barnes et al., 2002). Several viral components from different viruses have been shown to inhibit IFN-β production *via* interfering with IRF-3 signal pathway (Fensterl et al., 2005; Daffis et al., 2007; Paulmann et al., 2008). We therefore determined the effects of NTPase on IRF-3 signal pathway. PRD(III-I)_4_-Luc containing four repeats of the IRF-3 responsive domain of IFN-β promoter and phRL-TK together with NTPase-expressing plasmid or empty vector was cotransfected into HEK 293T cells for 24 h. Cells were subsequently stimulated with or without SeV for 16 h followed by DLR assay. The activation of IRF-3 responsive promoter was induced by SeV in cells transfected with the empty vector, while the activation was blocked by NTPase in a dose-dependent manner (Figure 3A). It is likely that IFN-β production was inhibited by NTPase through IRF-3 dependent signal pathway. Upon activation, IRF-3 phosphorylates and translocates from the cytoplasm to the nucleus, resulting in transcription induction of the gene encoding IFN-β (Goubau et al., 2013; Hartmann, 2017). We further verified the effect of NTPase on the phosphorylation and translocation of IRF-3. To analyze IRF-3 phosphorylation, cells transfected with plasmid expressing NTPase or empty vector were stimulated with SeV to activate IRF-3 signal pathway. As shown in Figure 3B, the phosphorylation of IRF-3 induced by SeV was inhibited by NTPase. Nuclear translocation of IRF-3 was analyzed in cells transfected with plasmid expressing NTPase or empty vector and stimulated with SeV to induce IRF-3 translocation from the cytoplasm to the nucleus. Cytoplasmic and nuclear proteins were subsequently isolated from HEK 293T cells followed by WB to determine the distribution of IRF-3. As shown in Figures 3C,D, IRF-3 nuclear translocation significantly decreased in cells transfected with NTPase expression plasmid. To further confirm the results, HeLa cells were transfected with NTPase expression plasmid or empty vector. At 24 h posttransfection, cells were stimulated with or without SeV for 16 h and examined by indirect immunofluorescence (IF). As shown in Figure 3E, after stimulation with SeV, IRF-3 in cells transfected with empty vector mostly located in the nucleus, whereas IRF-3 nuclear localization significantly decreased in cells transfected with NTPase expression plasmid. These data collectively indicate that HuNoV NTPase inhibits the activation of IRF-3.

### HuNoV NTPase Interrupts IFN-β Production at the Stage of TBK1/IKKε Kinases in IRF-3 Signal Pathway

**Figure 4:**
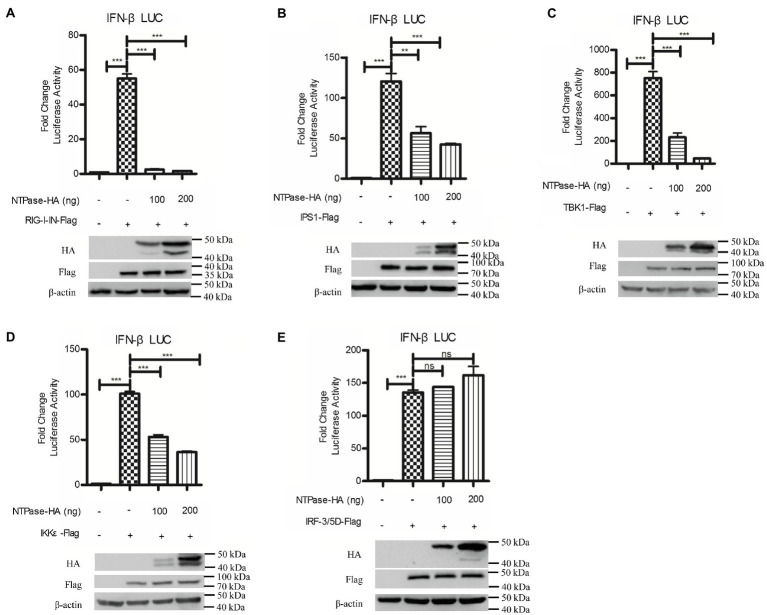
HuNoV NTPase blocks IRF-3 signaling at the stage of tANK-binding kinase 1 (TBK1)/IKKε kinase. **(A-E)** Various amounts of HA-tagged NTPase expression plasmid or empty vector together with 100 ng p125-Luc and 20 ng phRL-TK, together with 20 ng retinoic acid-inducible gene (RIG)-I-IN **(A)**, IPS1 **(B)**, TBK1 **(C)**, IKKε **(D)**, or IRF-3/5D **(E)** expression plasmid were cotransfected into HEK 293T cells preseeded in 48-well plates for 40 h. Cells were harvested and luciferase activities were measured with DLR assay. Protein expression was monitored by WB using anti-HA or anti-Flag Ab. β-actin was used as a loading control. One representative experiment out of three is shown for WB. For graphs, data shown are mean ± SD of three independent experiments, with each condition performed in triplicate. ^***^p < 0.001; ^**^p < 0.01; and ns, not significant.

It is known that HuNoV replication efficiently generates double-stranded RNA (Qu et al., 2016). RIG-I can bind to dsRNA and signal cascade through the adaptor IPS1 in response to foreign RNA (Esser-Nobis et al., 2020; Schweinoch et al., 2020). Two protein kinase complex, TBK1/IKKε is engaged to initiate RIG-I signal, leading to the phosphorylation and further activation of IRF-3 (Fitzgerald et al., 2003; Fang et al., 2017). To identify the potential mechanism by which NTPase inhibits IFN-β production, HEK 293T cells were cotransfected with p125-Luc, phRL-TK, NTPase expression plasmid, or empty vector, together with plasmid expressing RIG-I-IN, IPS1, TBK1, IKKε, or IRF-3/5D, which are inducers of IFN-β in the IRF-3 signal pathway. As shown in Figures 4A–E, overexpression of RIG-I-IN (Figure 4A), IPS1 (Figure 4B), TBK1 (Figure 4C), IKKε (Figure 4D), or IRF-3/5D (Figure 4E) directly induced the activation of IFN-β promoter, which is consistent with a previous study (Jaworska et al., 2007). As shown in Figures 4A–E, NTPase suppressed the activation of IFN-β promoter induced by RIG-I-IN, IPS1, TBK1, and IKKε in a dose-dependent manner, but had no effect on the activation of IFN-β promoter induced by IRF-3. These results indicate that NTPase inhibits the production of IFN-β at the stage of TBK1/IKKε kinases in IRF-3 signal pathway.

### HuNoV NTPase Inhibits IKKε Phosphorylation *via* a Physical Interaction

**Figure 5:**
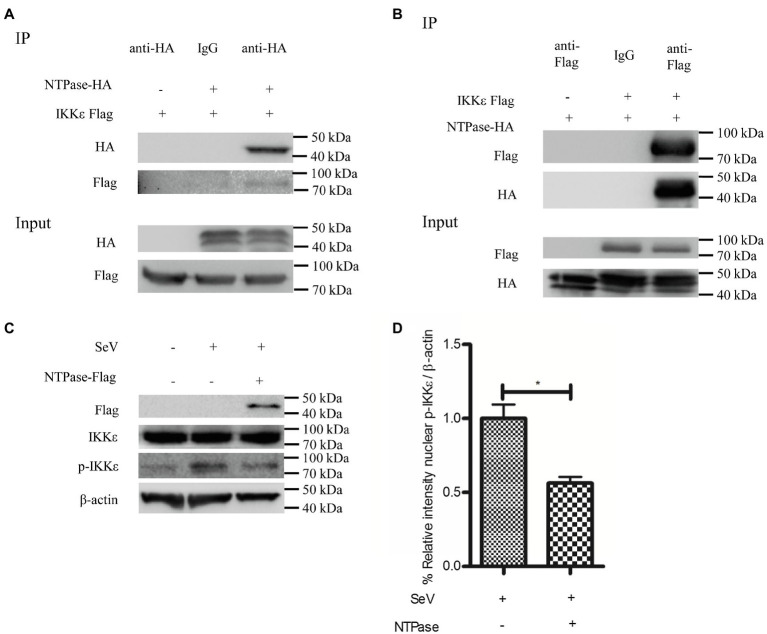
HuNoV NTPase interacts with IKKε and inhibits its phosphorylation. **(A,B)** HuNoV NTPase interacts with IKKε. **(A)** HEK 293T cells in 6-well plates were cotransfected with 1.5 μg plasmid expressing Flag-tagged IKKε together with 1.5 μg HA-tagged NTPase expression plasmid or empty vector for 40 h. Cells were lysed and subjected to coimmunoprecipitation (Co-IP) using rabbit anti-HA Ab. **(B)** HEK 293T cells in 6-well plates were cotransfected with 1.5 μg HA-tagged NTPase expression plasmid together with 1.5 μg plasmid expressing Flag-tagged IKKε or empty vector for 40 h. A mouse anti-Flag Ab was used for Co-IP of cell lysates with normal IgG being used as a negative control. IP products and 5% input samples were examined by WB using anti-HA and anti-Flag Abs. **(C)** HuNoV NTPase inhibits the phosphorylation of IKKε. HEK 293T cells in 6-well plates were transfected with 3 μg plasmid expressing NTPase or empty vector for 24 h and then stimulated with or without SeV for 16 h. Total protein was isolated. WB was performed to examine IKKε and p-IKKε using corresponding Abs. The expression of NTPase was monitored using anti-Flag Ab. **(D)** Blot intensity for p-IKKε was quantified using Image Lab with statistical significance being analyzed from three independent experiments. One representative experiment out of three is shown for WB. For graphs, data shown are mean ± SD of three independent experiments, with each condition performed in triplicate. ^*^p < 0.05.

IKKε and TBK1 are essential components of IRF-3 activation (Fitzgerald et al., 2003; tenOever et al., 2004; Fang et al., 2017). We hypothesized that NTPase may interfere with the function of TBK1 or IKKε *via* the interaction. To test this hypothesis, coimmunoprecipitation (Co-IP) was performed to determine the interaction between NTPase and TBK1 or IKKε. HEK 293T cells were cotransfected with plasmid expressing IKKε-Flag together with NTPase-HA expression plasmid or empty vector for 40 h. Precleared cell lysates from the transfected cells were incubated with the HA Ab against NTPase-HA. The precipitates were analyzed by WB using anti-Flag Ab against Flag-tagged IKKε. As shown in Figure 5A, the anti-HA Ab was able to specifically precipitate the immune complex that contained NTPase-HA and IKKε-Flag. To further confirm the physical association of NTPase with IKKε, plasmid expressing NTPase-HA together with IKKε-Flag expression plasmid or empty vector was cotransfected into HEK 293T cells for 40 h. Precleared cell lysates from the transfected cells were incubated with anti-Flag Ab against IKKε-Flag. The precipitates were analyzed by WB using anti-HA Ab against HA-tagged NTPase. As shown in Figure 5B, the anti-Flag Ab was able to specifically precipitate the immune complex that contained IKKε-Flag and NTPase-HA. The Co-IP results indicated that NTPase interacted with IKKε, but had no interaction with TBK1 (data not shown). We then asked whether NTPase affects the activation and function of IKKε. Phosphorylation of IKKε was analyzed in cells transfected with plasmid expressing NTPase or empty vector and stimulated with SeV to activate IRF-3 signal pathway. As expected, after the stimulation with SeV, IKKε phosphorylation was induced in cells transfected with empty vector but significantly decreased in cells transfected with NTPase expression plasmid (Figures 5C,D). These results indicate that the interaction of NTPase with IKKε likely results in the inhibition of IKKε phosphorylation.

### The 1-179 aa Domain of NTPase Is Important in Inhibiting IFN-β Production

**Figure 6:**
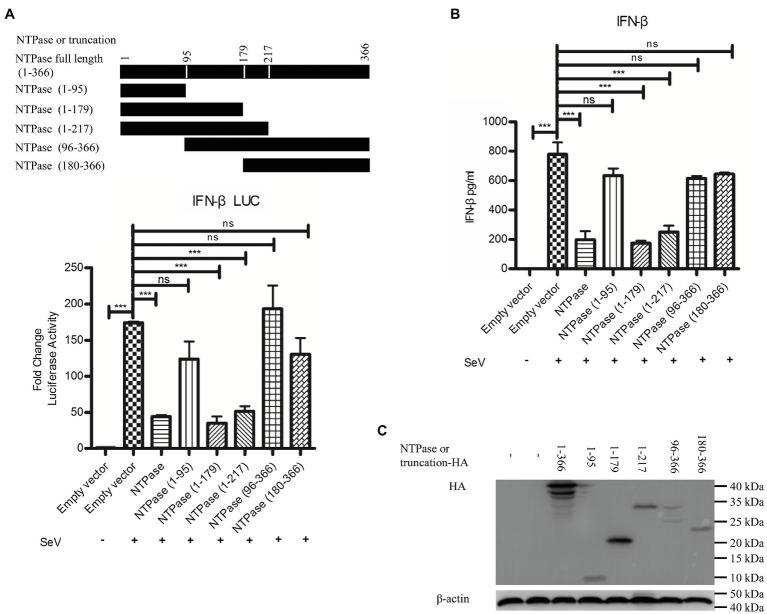
The 1-179 aa domain of NTPase is important in inhibiting IFN-β production. **(A)** The 1-179 aa and 1-217 aa domains of NTPase significantly inhibit the activation of IFN-β promoter. 200 ng NTPase, truncated NTPase expression plasmid, or empty vector together with 100 ng p125-Luc and 20 ng phRL-TK was cotransfected into HEK 293T cells preseeded in 48-well plates for 24 h followed by stimulation with or without SeV for 16 h. Reporter activities were determined with DLR assay. **(B)** The 1-179 aa and 1-217 aa domains of NTPase inhibit the production of IFN-β. 3 μg NTPase, truncated NTPase expression plasmid, or empty vector was transfected into HEK 293T cells preseeded in 6-well plates. At 24 h posttransfection, cells were stimulated with or without SeV for 16 h. The protein level of IFN-β in supernatants was measured by ELISA. **(C)** The expression of NTPase and truncated NTPase is determined by anti-HA Ab. One representative experiment out of three is shown for WB. For graphs, data shown are mean ± SD of three independent experiments, with each condition performed in triplicate. ^***^p < 0.001; ns, not significant.

Mitochondrion functions as a signaling platform for RLRs (Cloonan and Choi, 2013; Banoth and Cassel, 2018). Two mitochondrion targeting domains were identified in the C-terminal region (96-366 aa) of GII.4-NTPase (Yen et al., 2018). It is tempting to know whether NTPase-mediated inhibition of IFN-β production is related to its mitochondrial location. To map the functional region of NTPase involved in the inhibition of IFN-β production, we constructed five truncation mutants including NTPase (1-95 aa), NTPase (1-179 aa), NTPase (1-217 aa), NTPase (96-366 aa), and NTPase (180-366 aa) according to its subcellular localization and interaction domain (Yen et al., 2018). Plasmid expressing NTPase, truncated NTPase, or empty vector together with p125-luc and phRL-TK was cotransfected into HEK 293T cells. At 24 h posttransfection, cells were stimulated with or without SeV for 16 h. Reporter activities were determined with DLR assay. As seen in Figure 6A, the mutants with N terminal region deletion [NTPase (96-366 aa) and NTPase (180-366 aa)] barely suppressed the activation of IFN-β promoter induced by SeV, whereas the mutants with C terminal region deletion [NTPase (1-179 aa) and NTPase (1-217 aa)] significantly inhibited the activation of IFN-β promoter. We further confirm our findings generated from DLR assay at protein level. NTPase or its truncated mutant expression plasmid, or empty vector was transfected into HEK 293T cells. At 24 h posttransfection, cells were stimulated with or without SeV for 16 h. The IFN-β in supernatants was measured by ELISA. In agreement with the results generated from DLR assay, NTPase (1-179 aa) and NTPase (1-217 aa) but not NTPase (1-95 aa), NTPase (96-366 aa), or NTPase (180-366 aa) inhibited IFN-β production at protein level (Figure 6B). The expression of NTPase and truncated NTPase was confirmed as shown in Figure 6C. Taken together, these results indicate that the 1-179 aa domain of HuNoV NTPase is important in inhibiting IFN-β production.

### The 1-179 aa Domain of NTPase Inhibits the Production of IFN-β in the Same Manner as Full-Length NTPase

**Figure 7:**
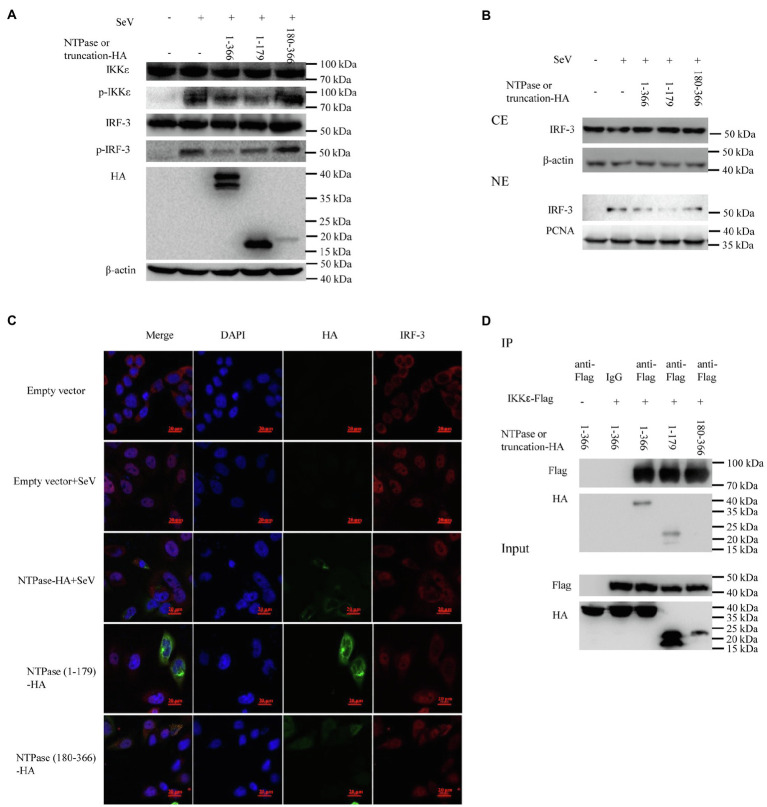
The 1-179 aa domain of NTPase inhibits IFN-β production in the same manner as full-length NTPase. **(A)** The 1-179 aa domain of NTPase inhibits the phosphorylation of IKKε and IRF-3 as does full-length NTPases. 3 μg plasmid expressing HA-tagged NTPase, truncated NTPase, or empty vector was transfected into HEK 293T cells preseeded in 6-well plates. At 24 h posttransfection, cells were stimulated with or without SeV for 16 h. Total protein was isolated. WB was performed to examine IKKε, p-IKKε, IRF-3, and p-IRF-3 using corresponding Abs. The expression of NTPase and the truncated NTPase was monitored using anti-HA Ab. **(B,C)** The 1-179 aa domain of NTPase inhibits IRF-3 nuclear translocation. **(B)** 3 μg NTPase, truncated NTPase expression plasmid, or empty vector was transfected into HEK 293T cells preseeded in 6-well plates for 24 h followed by stimulation with or without SeV for 16 h. Cytoplasmic and nuclear proteins were isolated. IRF-3 levels were measured with anti-IRF-3 Ab. β-actin and proliferating cell nuclear antigen were used as loading controls for cytoplasmic and nuclear proteins, respectively. **(C)** 3 μg HA-tagged NTPase, truncation expression plasmid, or empty vector was transfected into HeLa cells preseeded in 35-mm dishes. At 24 h posttransfection, cells were stimulated with or without SeV for 16 h. Cells were incubated with mouse anti-HA Ab and rabbit anti-IRF-3 pAb, followed by Alexa Fluor 488-labeled Goat Anti-Mouse IgG (H + L) (green) and Alexa Fluor 647-labeled Goat Anti-Rabbit IgG (H + L) (red) as the secondary Abs. Cell nuclei (blue) were stained with DAPI. The images were acquired by fluorescence microscopy using a 60 × objective. One representative experiment out of three is shown. **(D)** The 1-179 aa domain of NTPase interacts with IKKε as does full-length NTPases. HEK 293T cells in 6-well plates were cotransfected with 1.5 μg HA-tagged NTPase or truncated NTPase expression plasmid together with 1.5 μg plasmid expressing Flag-tagged IKKε or empty vector for 40 h. A mouse anti-Flag Ab was used for Co-IP of cell lysates with normal IgG being used as a negative control. Co-IP products and 5% input samples were examined using rabbit anti-HA and mouse anti-Flag Abs with WB. One representative experiment out of three is shown for WB. CE, cytoplasmic protein extract; NE, nuclear protein extract.

Because NTPase inhibits IFN-β production by inhibiting IRF-3 activation *via* targeting IKKε, the 1-179 aa domain of NTPase likely interferes with IFN-β production in the same manner as full-length NTPase. Subsequent experiments were conducted to address the mechanism of NTPase (1-179 aa) suppressing IFN-β production. We firstly analyzed the phosphorylation of IKKε and IRF-3 in cells transfected with NTPase or truncated NTPase expression plasmid or empty vector followed by stimulation with SeV to activate IRF-3 signal pathway. As shown in Figure 7A, NTPase and NTPase (1-179 aa) but not NTPase (180-366 aa) inhibited the phosphorylation levels of IKKε and IRF-3 induced by SeV. Of interest, the truncation mutant NTPase (1-179 aa) appeared to inhibit IKKε phosphorylation in a stronger manner than did the full-length NTPase. HEK 293T cells transfected with plasmid expressing NTPase, truncated NTPase, or empty vector for 24 h were stimulated with SeV for 16 h to induce IRF-3 translocation from the cytoplasm to the nucleus. Cytoplasmic and nuclear proteins were subsequently isolated from HEK 293T cells, followed by WB to determine the distribution of IRF-3. NTPase and NTPase (1-179 aa) but not NTPase (180-366 aa) significantly inhibited IRF-3 nuclear translocation. Moreover, the truncation mutant NTPase (1-179 aa) appeared to inhibit the nuclear translocation of IRF-3 more robustly than did the full-length NTPase (Figure 7B). To further confirm the results, HeLa cells were transfected with NTPase expression plasmid, truncated NTPase expression plasmid, or empty vector for 24 h followed by stimulation with or without SeV for 16 h. Cells were examined by indirect IF. As shown in Figure 7C, under SeV stimulation, in comparison with cells transfected with empty vector, nuclear IRF-3 decreased in cells transfected with NTPase or NTPase (1-179 aa) expression plasmid but not those with NTPase (180-366 aa) expression plasmid. Plasmid expressing NTPase-HA or truncated NTPase-HA together with IKKε-Flag expression plasmid or empty vector was cotransfected into HEK 293T cells for 40 h. Precleared cell lysates from the transfected cells were incubated with anti-Flag Ab against IKKε-Flag, and the precipitates were analyzed by WB using anti-HA Ab against HA-tagged NTPase. As shown in Figure 7D, the anti-Flag Ab was able to specifically precipitate the immune complex that contained IKKε, NTPase, and NTPase (1-179 aa), but not NTPase (180-366 aa). These results together suggest that the 1-179 aa domain inhibits IFN-β production in the same manner as the full-length NTPase and that the mutant NTPase (1-179 aa) inhibits IRF-3 nuclear translocation and IKKε phosphorylation more robustly than does the full-length NTPase.

### HuNoV NTPase and 1-179 aa Domain Block the Association of IKKε With Unanchored K48-Linked Polyubiquitin Chains

**Figure 8:**
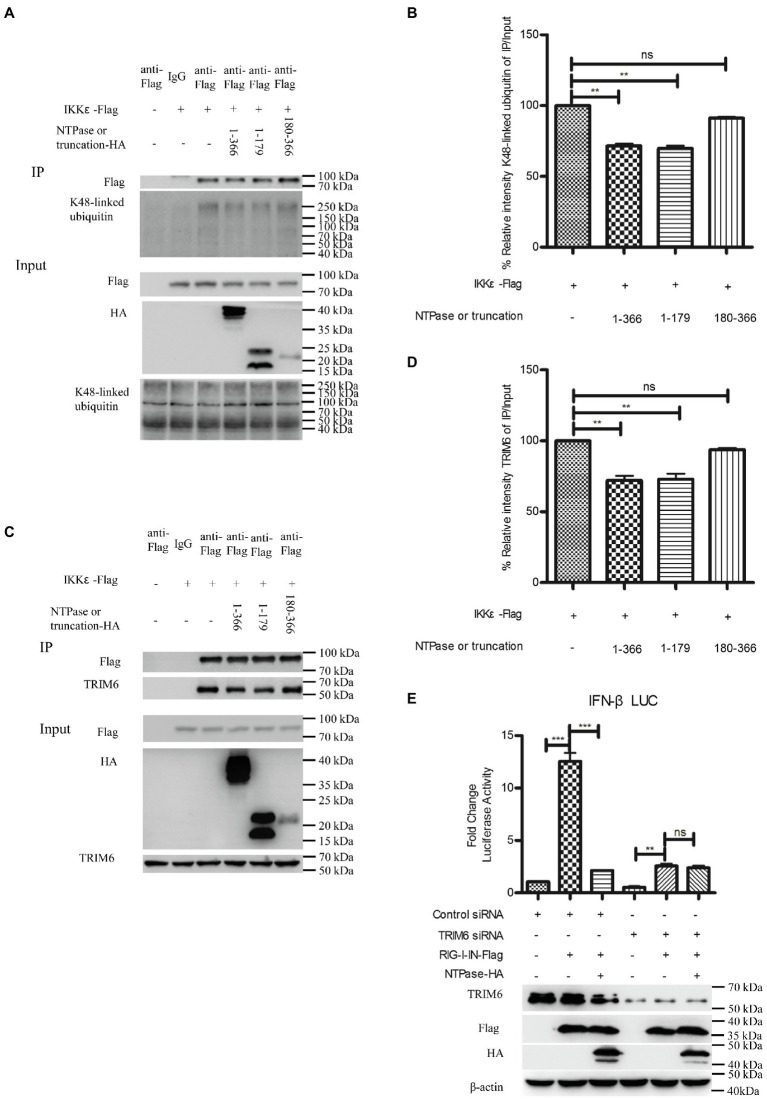
HuNoV NTPase reduces the association of IKKε with unanchored K48-linked polyubiquitin chains and tripartite motif-containing 6 (TRIM6). **(A)** HuNoV NTPase suppresses the interaction of IKKε with K48-linked polyubiquitin chains. HEK 293T cells in 6-well plates were cotransfected with 1.5 μg plasmid expressing HA-tagged NTPase or truncated NTPase together with 1.5 μg Flag-tagged IKKε expression plasmid or empty vector for 40 h. A mouse anti-Flag Ab was used for Co-IP of cell lysates with normal IgG being used as a negative control. 5% input samples were examined by WB using anti-HA Ab, anti-Flag Ab, and anti-K48-linked polyubiquitin Ab. IP products were examined by WB using anti-Flag Ab and anti-K48-linked polyubiquitin Ab. **(B)** Blot intensity for K48-linked polyubiquitin which interacted with IKKε was quantified using Image Lab with statistical significance being analyzed from three independent experiments. **(C)** HuNoV NTPase reduces the interaction of IKKε with TRIM6. HEK 293T cells in 6-well plates were cotransfected with 1.5 μg plasmid expressing HA-tagged NTPase or truncated NTPase together with 1.5 μg Flag-tagged IKKε expression plasmid or empty vector for 40 h. A mouse anti-Flag Ab was used for Co-IP of cell lysates with normal IgG being used as a negative control. 5% input samples were examined by WB using anti-HA Ab, anti-Flag Ab, and anti-TRIM6 Ab. IP products were examined by WB using anti-Flag Ab and anti-TRIM6 Ab. **(D)** Blot intensity for TRIM6 which interacted with IKKε was quantified using Image Lab with statistical significance being analyzed from three independent experiments. **(E)** The IFN-β induction was not affected by NTPase with TRIM6 knockdown. HEK 293T cells in 48-well plates were transfected with 5 nm siRNA against TRIM6 or control siRNA. 24 h later, cells were cotransfected with 200 ng of HA-tagged NTPase expression plasmid or empty vector together with 100 ng p125-Luc and 20 ng phRL-TK, together with 20 ng RIG-I-IN expression plasmid for another 24 h. Reporter activities were determined with DLR assay. The expression of TRIM6, NTPase, and RIG-I-IN was determined with anti-TRIM6 Ab, anti-HA Ab, and anti-Flag Ab, respectively. One representative experiment out of three is shown for WB. For graphs, data shown are mean ± SD of three independent experiments, with each condition performed in triplicate. ^***^p < 0.001; ^**^p < 0.01; and ns, not significant.

Ubiquitination of IKKε is involved in the control of its kinase activation and is required for inducing IRF-3 phosphorylation and nuclear localization to activate IFN-β promoter (Ikeda et al., 2007). Unanchored K48-linked polyubiquitin chains interact with IKKε and promote its oligomerization and autophosphorylation, leading to the activation of IKKε (Rajsbaum et al., 2014b; Bharaj et al., 2016). Subsequent experiments were conducted to examine whether NTPase suppresses the interaction of IKKε with K48-linked polyubiquitin chains. Plasmid expressing NTPase-HA or truncated NTPase-HA together with IKKε-Flag expression plasmid or empty vector was cotransfected into HEK 293T cells for 40 h. Precleared cell lysates from the transfected cells were incubated with anti-Flag Ab against IKKε-Flag. The precipitates were analyzed by WB using anti-K48-linked polyubiquitin. As shown in Figures 8A,B, the anti-Flag Ab against IKKε-Flag was able to specifically precipitate the immune complex that contained IKKε and K48-linked unanchored polyubiquitin chains, but K48-linked unanchored polyubiquitin chains of the precipitation significantly decreased in NTPase- or its truncation 1-179 aa-transfected cells. TRIM6, a member of the TRIM E3-ubiquitin ligase family, associates with IKKε and catalyzes the synthesis of unanchored K48-linked polyubiquitin chains (Rajsbaum et al., 2014b; Bharaj et al., 2016). We performed additional experiments to confirm whether NTPase blocks the association of IKKε with TRIM6. As shown in Figures 8C,D, the anti-Flag Ab against IKKε-Flag was able to specifically precipitate the immune complex that contained IKKε and TRIM6, and the precipitated TRIM6 decreased in NTPase- or its truncation 1-179 aa- but not 180-366 aa-transfected cells. To test whether K48-linked polyubiquitin chains synthesized by TRIM6 have functional relevance, we examined the ability of NTPase in inhibiting IFN-β promoter activation induced by RIG-I-IN in TRIM6 knockdown cells. TRIM6-specific siRNA or control siRNA was transfected into HEK 293T cells. At 24 h posttransfection, cells were cotransfected with NTPase expression plasmid or empty vector, reporter plasmid p125-Luc and phRL-TK, together with plasmid encoding IFN-β inducer RIG-I-IN for another 24 h. The enzymatic activities of firefly and renilla luciferase were measured. As shown in Figure 8E, the activation of IFN-β promoter induced by RIG-I-IN was markedly inhibited in TRIM6 siRNA-transfected cells, although the activation was still detectable, in agreement with a previous report (Rajsbaum et al., 2014b). As expected, this residual IFN-β induction was not significantly affected by exogenous NTPase expression with TRIM6 knockdown. The expression of TRIM6, NTPase, and RIG-I-IN was confirmed by WB. These results together indicate that HuNoV NTPase likely interferes with the interaction between IKKε and K48-linked polyubiquitin chains synthesized by the TRIM6, which results in the inhibition of IKKε, leading to the suppression of IFN-β production.

## Discussion

Although HuNoV infection in the gnotobiotic pig model and HIEs was shown to be controlled in the presence of IFN-β, HuNoV itself did not induce IFN-β expression (Souza et al., 2007; Jung et al., 2012; Lin et al., 2019, 2020; Hosmillo et al., 2020), implying that HuNoV may inhibit the production of IFN-β to evade the host immunity. Due to the lack of an efficient *in vitro* culture system to perform experiments in the context of viral infection, HuNoV cDNA clone was analyzed as an alternative. It was reported that GII.3 HuNoV cDNA clone, which efficiently generates double-stranded RNAs, did not induce detectable type I IFN responses but rather inhibited IFN-β production (Qu et al., 2016). In this study, we showed that a cDNA clone of the most prevalent HuNoV GII.4 genotype inhibited IFN-β production. Furthermore, HuNoV NTPase was shown to inhibit not only the promoter activity of IFN-β but also the mRNA and protein production of IFN-β. In the condition of NTPase knockdown with siRNA, the HuNoV cDNA clone was shown to induce IFN-β production (Supplementary Figure S2), indicating that NTPase may be a key viral component of HuNoV responsible for inhibiting IFN-β production.

IRF-3 signal pathway is known to play a critical role in viral and bacterial innate immune responses by regulating IFN-β production (Sato et al., 1998; Barnes et al., 2002). Several viral components from different viruses have been shown to inhibit IFN-β production *via* interfering with IRF-3 signal pathway (Fensterl et al., 2005; Daffis et al., 2007; Paulmann et al., 2008). In this study, we found that NTPase inhibited the activity of IRF-3-responsive promoter. Upon activation, IRF-3 phosphorylates and translocates from the cytoplasm to the nucleus, resulting in transcription induction of the gene encoding IFN-β (Sharma et al., 2003). HuNoV NTPase in this study was demonstrated to reduce the phosphorylation of IRF-3 and thereby inhibit IRF-3 localization to the nucleus.

For RNA viruses, the major sensors RIG-I and MDA5 bind the adaptor molecule IPS1 upon virus infection, which activates TBK1 and IKKε, resulting in IRF-3 phosphorylation (Chiang et al., 2014; Kell and Gale, 2015). By assessing these IFN-β inducers in the IRF-3 signal pathway, we found that NTPase suppressed RIG-I-IN-, IPS1-, TBK1-, or IKKε but not IRF-3-induced activation of IFN-β promoter in a dose-dependent manner, suggesting that NTPase inhibits IFN-β production probably at the stage of TBK1/IKKε kinases in IRF-3 signal pathway. It is known that IKKε is important for IRF-3 phosphorylation (Fitzgerald et al., 2003; Fang et al., 2017). Several viruses have been shown to inhibit IRF-3 activation by targeting IKKε (Pythoud et al., 2012; Bharaj et al., 2016; Lundberg et al., 2019; Fang et al., 2020; Wong et al., 2020). Indeed, HuNoV NTPase was demonstrated to interact with IKKε and reduce its phosphorylation. We revealed that HuNoV NTPase inhibits the activation of IRF-3 by targeting IKKε.

Ubiquitination of IKKε is required to induce IRF-3 phosphorylation and nuclear localization, resulting in the activation of IFN-β promoter (Ikeda et al., 2007). Unanchored K48-linked polyubiquitin chains which are synthetized by the E3-ubiquitin ligase TRIM6, interact with IKKε, and promote IKKε activation (Versteeg et al., 2013; Rajsbaum et al., 2014a,b). It is known that Nipah Virus M targets TRIM6 to inhibit K48-linked polyubiquitin chains, leading to the inhibition of IKKε activation (Bharaj et al., 2016). In our study, we found that HuNoV NTPase suppressed the interaction of IKKε with unanchored K48-linked polyubiquitin and TRIM6. However, IFN-β induction was not significantly affected by exogenous NTPase expression with TRIM6 knockdown. These findings together indicate that the interaction of NTPase with IKKε likely blocks the interaction of IKKε with K48-linked polyubiquitin chains synthetized by TRIM6, leading to the inhibition of IKKε activation.

In the case of IFN-I signaling, both STAT1 and STAT2 are activated, leading to the formation of heterodimers that associate with the IRF-9 to form a transcription factor complex, termed IFN-stimulated gene factor 3 (Levy et al., 1989; Fu et al., 1990). In contrast, IFN-II (IFN-γ) signaling results in the recruitment and tyrosine phosphorylation of two STAT1 proteins, leading to the formation of STAT1:STAT1 homodimers, a complex referred to as the gamma-activated factor (Shuai et al., 1992). The activated IKKε phosphorylates STAT1 and inhibits its homodimerization, thereby regulating the balance between type I and type II IFN responses (Ng et al., 2011). As NTPase was determined to inhibit IKKε activation, we speculated that NTPase may regulate type I and type II IFN signal pathway. Indeed, we found that NTPase inhibited the expression of MDA5 and STAT1 induced by IFN-β but promoted the expression of IRF-1 and STAT1 induced by IFN-γ (Supplementary Figure S3). These may explain why GII.4 HuNoV induces ISGs through type II IFN pathways depending on STAT1 (Lin et al., 2020).

Mitochondrion functions as a signaling platform for RLRs (Cloonan and Choi, 2013; Banoth and Cassel, 2018). Two mitochondrion targeting domains were identified in the C-terminal region (96-366 aa) of GII.4-NTPase (Yen et al., 2018). It is tempting to know whether NTPase-mediated inhibition of IFN-β production is related to its mitochondrial location. We showed that NTPase 96-366 aa region had no impact on the production of IFN-β, implying that NTPase inhibits IFN-β production regardless of its mitochondria location. The N-terminal 1-179 aa region of GII.4 HuNoV NTPase was revealed to be sufficient in mediating the formation of homodimers or homo-oligomers and the interaction of NTPase with two other nonstructural proteins p48 and p22 (Yen et al., 2018). In our study, NTPase was shown to inhibit IFN-β production by interacting with IKKε. It is likely the 1-179 aa region of NTPase is responsible for inhibiting IFN-β production. In agreement, we reveled that NTPase 1-179 aa and 1-271 aa region but not 1-95 aa or 180-366 aa region inhibited IFN-β production. The 1-179 aa domain of NTPase inhibited IFN-β production in the same manner as its full-length *via* interacting with IKKε. These findings strongly suggest that the function domain (1-179 aa) is important for NTPase-mediated inhibition of IFN-β production. HuNoV NTPase plays an important role in RNA synthesis *in vitro* (Pfister and Wimmer, 2001; Han et al., 2018; Li et al., 2018) and contains motifs A (GI HuNoV NTPase 162-169 aa), B (GI HuNoV NTPase 212 and 213 aa), and C (257-260 aa) relating to NTPase activity (Liu et al., 1996; Li et al., 2018). Mutations in motif A, B, or C abolished NTPase activity (Pfister and Wimmer, 1999). We found that the 1-179 aa domain of NTPase containing motif A but not motif B or C is crucial for inhibiting IFN-β production. All these together suggest that HuNoV NTPase inhibits IFN-β production independent of its NTPase activity.

In summary, we report here that the cDNA clone of GII.4 HuNoV inhibits IFN-β production and its NTPase plays a critical role in the process. We reveal that HuNoV NTPase inhibits IFN-β production by inhibiting the phosphorylation and nuclear translocation of IRF-3. We further demonstrate that HuNoV NTPase interacts with IKKε and such interaction likely blocks the interaction of IKKε with the unanchored K48-linked polyubiquitin chains synthesized by TRIM6, resulting in inhibition of IKKε activation. We provide evidence that the 1-179 aa domain of NTPase is the functional region to interrupt IFN-β production. Our findings highlight the significance of GII.4 HuNoV NTPase in suppressing IFN-β production, providing a novel mechanism underlying how HuNoV evades the host innate immunity.

## Data Availability Statement

The original contributions presented in the study are included in the article/Supplementary Material, and further inquiries can be directed to the corresponding authors.

## Author Contributions

QH and SG supervised the research. ZZ and QH conceived the study. ZZ and YLi performed the experiments. ZZ and YLiu analyzed the data. ZZ, MF, and MZ provided the reagents and technical assistance and contributed to completion of the study. ZZ drafted the manuscript. QH reviewed and finalized the manuscript. All authors reviewed the results and approved the final version of the manuscript.

### Conflict of Interest

The authors declare that the research was conducted in the absence of any commercial or financial relationships that could be construed as a potential conflict of interest.

## Abbreviations

IFN: interferon
HuNoV: human norovirus
IKKε: IkB kinase ε
IRF-3: interferon-regulatory factor-3
HIEs: human intestinal enteroids
SeV: Sendai virus
Poly(I:C): polyinosinic-polycytidylic acid
siRNA: small interfering RNA
ISG: interferon-stimulated gene
RIG-I: retinoic acid-inducible gene I
MDA5: melanoma differentiation-associated protein 5
RLR: RIG-I-like receptor
ORF: open reading frame
MAVS: mitochondrial antiviral signal
TBK1: TANK-binding kinase 1
Ab: antibody
PCNA: proliferating cell nuclear antigen
TRIM6: tripartite motif-containing 6
HAU: hemagglutinating unit
Co-IP: coimmunoprecipitation
DLR: dual luciferase report
WB: Western blotting
IF: immunofluorescence
