# Supplementary material for: Human Norovirus NTPase Antagonizes Interferon-β Production by Interacting With IkB Kinase ε
Source: Front Microbiol. 2021 Jul 15;12:687933. doi: 10.3389/fmicb.2021.687933 (PMC8319745; doi:10.3389/fmicb.2021.687933)
Supplement: Supplementary file 1 [file Data_Sheet_1.doc]

**
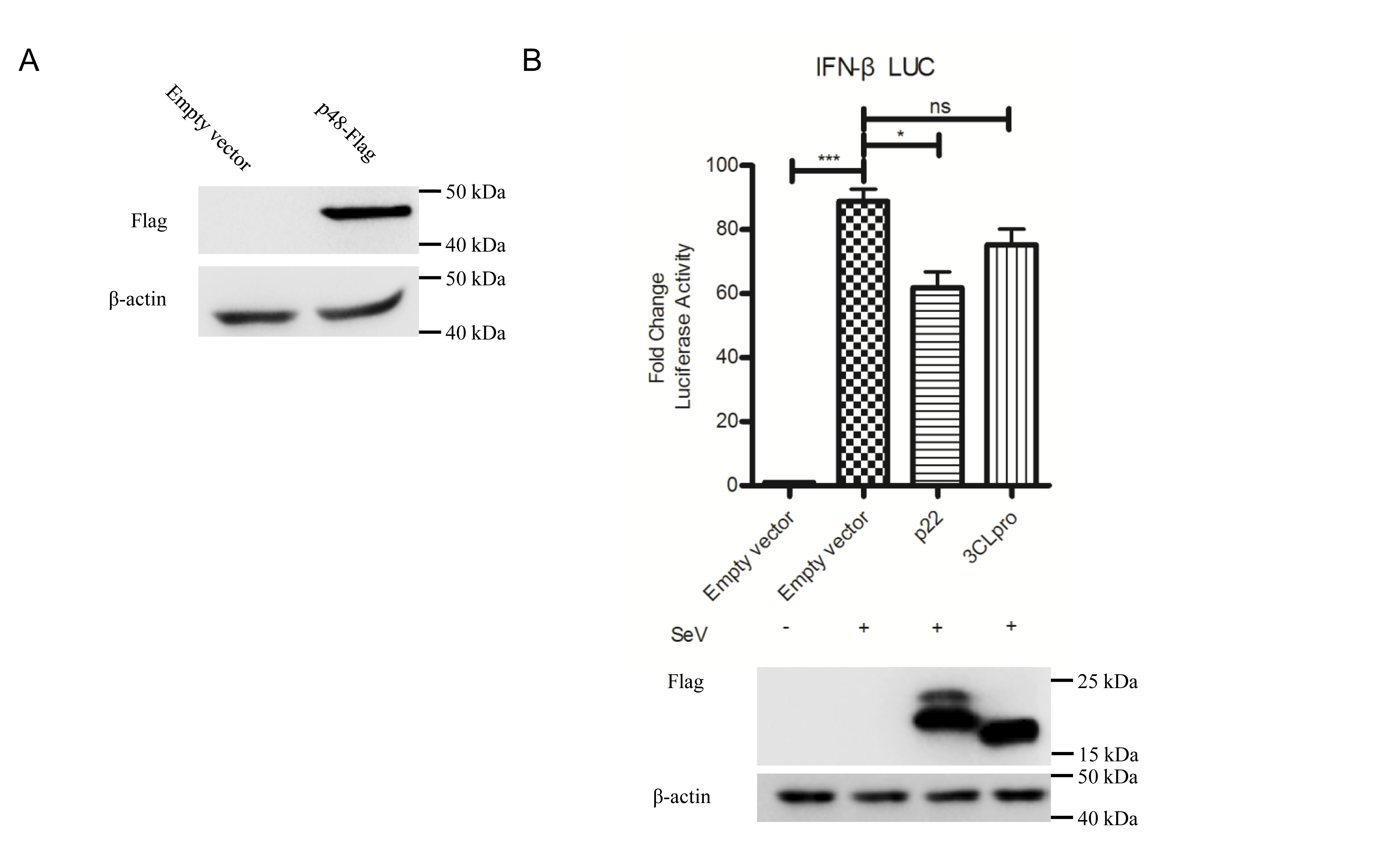
**

**FIGURE S1.** Identification of HuNoV proteins that inhibit IFN-β expression. (A) Confirmation of HuNoV p48 expression. 3 μg plasmid expressing Flag-tagged p48 or empty vector was transfected into HEK 293T cells for 24 h. The expression of p48 was detected by WB using an anti-Flag Ab. (B) Identification of HuNoV proteins that inhibit IFN-β expression. 200 ng plasmid expressing Flag-tagged p22, 3CLpro, or empty vector together with 100 ng p125-Luc and 20 ng phRL-TK were cotransfected into HEK 293T cells preseeded in 48-well plates for 24 h. Cells were stimulated with or without SeV for 16 h. DLR assay was performed to determine reporter activities. The expression of p22 and 3CLpro was analyzed by WB using an anti-Flag Ab. One representative experiment out of three is shown for WB. For graphs, data shown are mean ± SD of three independent experiments, with each condition performed in triplicate. ***p <0.001; *p <0.05; ns, not significant.


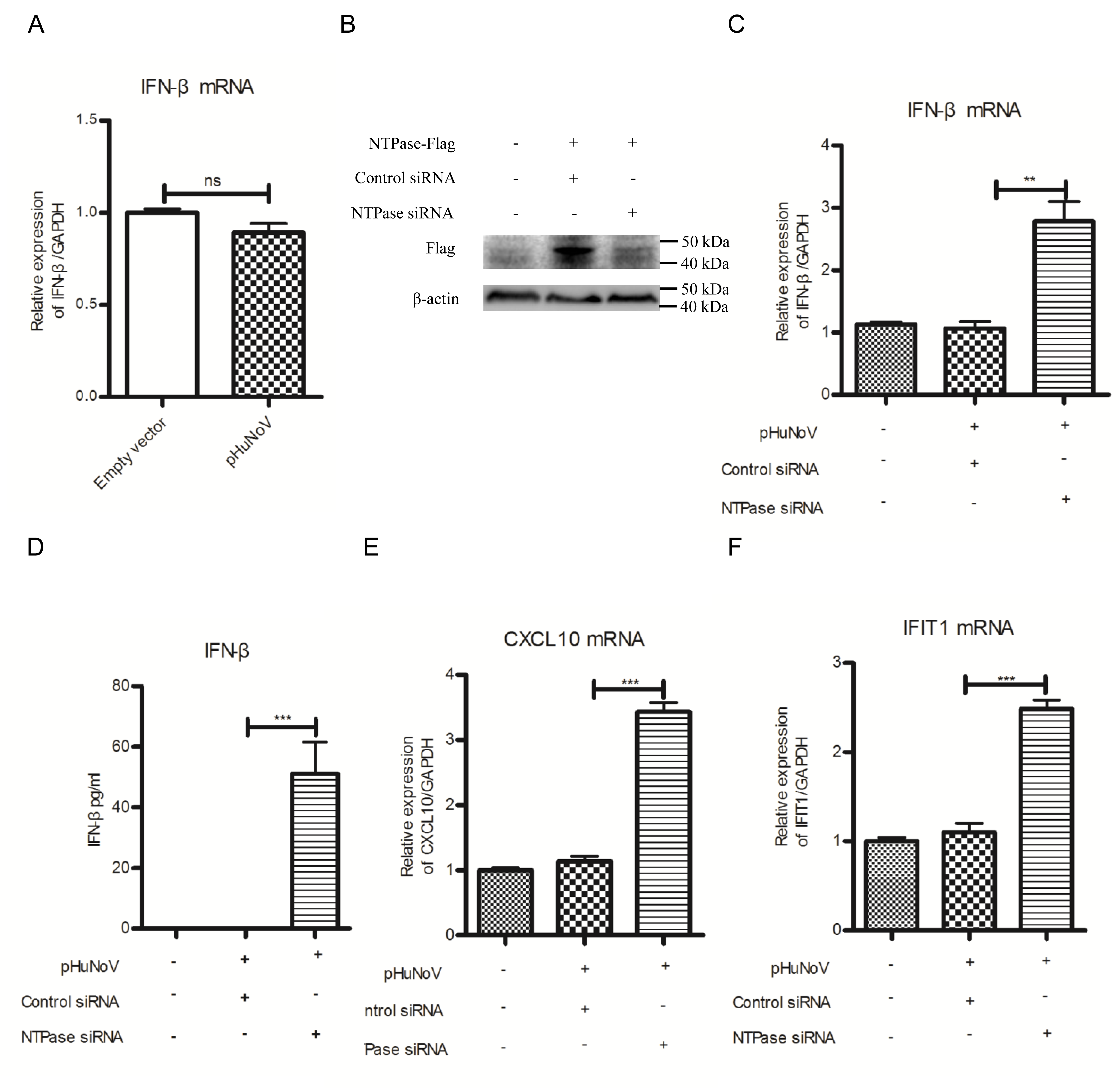


**FIGURE S2.** The HuNoV cDNA clone with NTPase knock-down increases the expression of IFN-β and ISGs. **(A)** The HuNoV cDNA clone has no effect on the expression of IFN-β. Caco-2 cells in 6-well plates were transfected with 3 μg pHuNoV or empty vector for 24 h. Total RNA was extracted from transfected cells to measure IFN-β mRNA by real-time PCR. **(B)** Knock-down efficiency of NTPase siRNA. Caco-2 cells in 6-well plates were transfected with 5 nM siRNA against NTPase or control siRNA. 24 h later, cells were transfected with 3 μg NTPase-Flag expression plasmid for another 24 h. Total protein was isolated. The expression of NTPase was monitored using anti-Flag Ab. **(C, D)** The HuNoV cDNA clone with NTPase knock-down increases the production of IFN-β mRNA and protein. Caco-2 cells in 6-well plates were transfected with 5 nM siRNA against NTPase or control siRNA. 24 h later, cells were transfected with 3 μg pHuNoV or empty vector for another 24 h. Total RNA was extracted from transfected cells to measure IFN-β mRNA by real-time PCR **(C)**, while IFN-β released in supernatants was determined by ELISA **(D)**. **(E, F)** The HuNoV cDNA clone with NTPase knock-down increases the expression of ISGs. Caco-2 cells in 6-well plates were transfected with 5 nM siRNA against NTPase or control siRNA. 24 h later, cells were transfected with 3 μg pHuNoV or empty vector for another 24 h. Total RNA was extracted from transfected cells to measure CXCL10 **(E)** and IFIT1 **(F)** mRNA by real-time PCR. For WB, one representative experiment out of three is shown. For graphs, data shown are mean ± SD of three independent experiments, with each condition performed in triplicate. ***p <0.001; **p <0.01; ns, not significant.


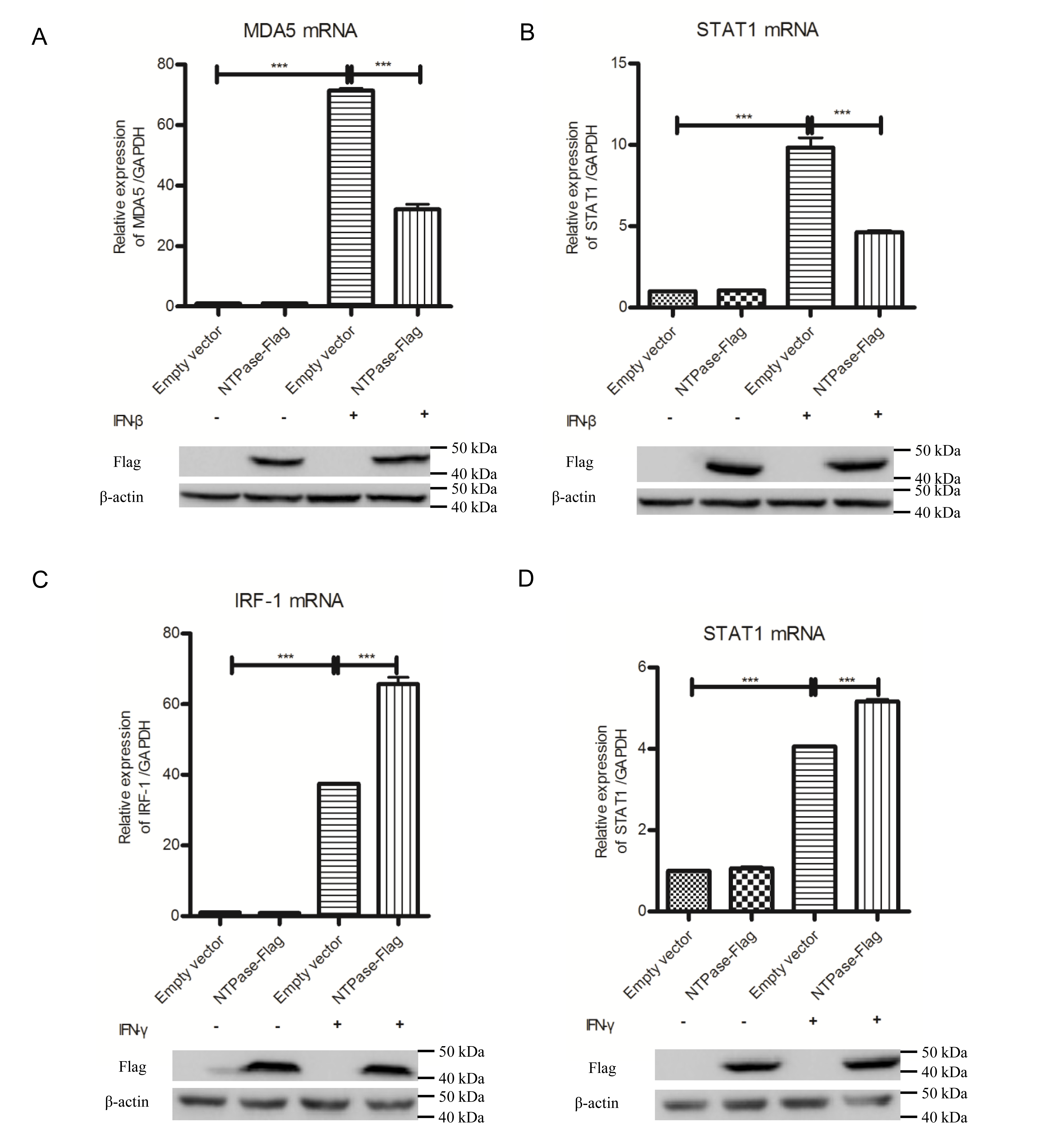


**FIGURE S3.** HuNoV NTPase inhibits ISGs mRNA expression induced by IFN-β but promotes ISGs mRNA expression induced by IFN-γ. Caco-2 cells in 6-well plates were transfected with 3 μg Flag-tagged NTPase expression plasmid. At 24 h posttransfection, cells were stimulated with IFN-β **(A, B)** or IFN-γ **(C, D)** for 16 h. Total RNA was extracted from transfected cells to measure MDA5 **(A)**, STAT1 **(B, D)** and IRF-1 **(C)** mRNAs by real-time PCR. The expression of HuNoV NTPase was detected using an anti-Flag Ab, with β-actin being used as a loading control. For WB, one representative experiment out of three is shown. For graphs, data shown are mean ± SD of three independent experiments, with each condition performed in triplicate. ***p <0.001.

Table S1

| **Primers for plasmid construction** | |
| --- | --- |
| **Primer name** | **Sequence** |
| **p48-F** | 5'-GTTTAAACTTAAGCTATGGATTACAAGGATGACGACGATAAGATGAAGATGGCGTCTAACGACG-3' |
| **p48-R** | 5'-CTGGACTAGTGGATCCTACTTATCGTCGTCATCCTTGTAATCTTGCAGTTCATAATCACCTAGCAAG-3' |
| **NTPase-Flag-F** | 5'-GTTTAAACTTAAGCTATGGGACCTGAGGACCTTGCAGTG-3' |
| **NTPase-Flag-R** | 5'-CTGGACTAGTGGATCCTACTTATCGTCGTCATCCTTGTAATCTTGCAGTTCATATTCATCTAGCCTC-3' |
| **NTPase-HA-F** | 5'-GTTTAAACTTAAGCTATGTACCCATACGACGTCCCAGACTACGCTGGACCTGAGGACCTTGCAGTG-3' |
| **NTPase-HA-R** | 5'-CTGGACTAGTGGATCCTAAGCGTAGTCTGGGACGTCGTATGGGTATTGCAGTTCATATTCATCTAGCCTC-3' |
| **VPg-F** | 5'-GTTTAAACTTAAGCTATGGATTACAAGGATGACGACGATAAGGGCAAGAAAGGGAAGAACAAC-3' |
| **VPg-R** | 5'-CTGGACTAGTGGATCCTACTCAAAGTTGAGTTTCTCACTGTAGTC-3' |
| **RdRP-F** | 5'-GTTTAAACTTAAGCTATGGATTACAAGGATGACGACGATAAGGGAGGTGACAGCAAAGGGACATACTG-3' |
| **RdRP-R** | 5'-CTGGACTAGTGGATCTCACTCGACGCCATCTTCATTCAC-3' |
| **VP1-F** | 5'-GTTTAAACTTAAGCTATGAAGATGGCGTCGAATGA-3' |
| **VP1-R** | 5'-CTGGACTAGTGGATCCTACTTATCGTCGTCATCCTTGTAATCCTTATCGTCGTCATCCTTGTAATCCTTATCGTCGTCATCCTTGTAATCTAATGCACGTCTACGCC-3' |
| **VP2-F** | 5'-GTTTAAACTTAAGCTATGGCTGGAGCTTTCTTTGC-3' |
| **VP2-R** | 5'-CTGGACTAGTGGATCCTACTTATCGTCGTCATCCTTGTAATCCTTATCGTCGTCATCCTTGTAATCCTTATCGTCGTCATCCTTGTAATCTGCCCGTGACTCCCCTC-3' |
| **NTPase (1-96)-R** | 5'-CTGGACTAGTGGATCCTAGAGTAGGGTGGTCATGTGGTTG-3' |
| **NTPase (1-179)-R** | 5'-CTGGACTAGTGGATCCTAGGCCAGTTCCCTGGCAAGGTG-3' |
| **NTPase (1-271)-R** | 5'-CTGGACTAGTGGATCCTAATAATCCAGTGGTGCTGGGTTG-3' |
| **NTPase (180-366)-F** | 5'-GTTTAAACTTAAGCTATGTACCCATACGACGTCCCAGACTACGCTAAGAAGATCGCGGCCTCCCTC-3' |

Table S2

| **Primers for real time PCR** | |
| --- | --- |
| **Primer name** | **Sequence** |
| **IFN-β-RT-F** | 5'-CAAATTGCTCTCCTGTTGTGCTTC-3' |
| **IFN-β-RT-R** | 5'-AATGCGGCGTCCTCCTTCT-3' |
| **NTPase-RT-F** | 5'-ACTGGCTATGGTGAGATCCATTGAGG-3' |
| **NTPase-RT-R** | 5'-ATCGGGTGAAGCAGACTTGGTTGAG-3' |
| **IFIT1-RT-F** | 5'-CCTCCTTGGGTTCGTCTACA-3' |
| **IFIT1-RT-F** | 5'-GGCTGATATCTGGGTGCCTA-3' |
| **IRF-1-RT-F** | 5'-TCCGGAGCTGGGCCATTCAC-3' |
| **IRF-1-RT-R** | 5'-GCTGAGCTGCCCTTGTTCC-3' |
| **CXCL10-RT-F** | 5'-GCTCTACTGAGGTGCTATGTTC-3' |
| **CXCL10-RT-R** | 5'-GGAGGATGGCAGTGGAAGTC-3' |
| **MDA5-RT-T** | 5'-GGCACCATGGGAAGTGATT-3' |
| **MDA5-RT-R** | 5'-ATTTGGTAAGGCCTGAGCTG-3' |
| **STAT1-RT-F** | 5'-CAGCTTGACTCAAAATTCCTGGA-3' |
| **STAT1-RT-R** | 5'-TGAAGATTACGCTTGCTTTTCCT-3' |
| **GAPDH-RT-F** | 5'-AGGGGCCATCCACAGTCTTC-3' |
| **GAPDH-RT-R** | 5'-AGAAGGCTGGGGCTCATTTG-3' |
